# Online Communities as a Support System for Alzheimer Disease and Dementia Care: Large-Scale Exploratory Study

**DOI:** 10.2196/68890

**Published:** 2025-05-05

**Authors:** Sidharth Kaliappan, Chunyu Liu, Yoshee Jain, Ravi Karkar, Koustuv Saha

**Affiliations:** 1 Manning College of Information & Computer Sciences University of Massachusetts Amherst Amherst, MA United States; 2 School of Information Sciences University of Illinois Urbana-Champaign Champaign United States; 3 Siebel School of Computing and Data Science The Grainger College of Engineering University of Illinois Urbana-Champaign Urbana, IL United States

**Keywords:** social media, natural language, Alzheimer disease, social support, online communities, machine learning

## Abstract

**Background:**

Alzheimer disease (AD) is the leading type of dementia, demanding comprehensive understanding and intervention strategies. In the United States, where over 6 million people are impacted, the prevalence of AD and related dementias (AD/ADRD) presents a growing public health challenge. However, individuals living with AD/ADRD and their caregivers frequently express feelings of marginalization, describing interactions characterized by perceptions of patient infantilization and a lack of respect.

**Objective:**

This study aimed to address 2 key research questions (RQs). For RQ1, we investigated the needs and concerns expressed by participants in online social communities focused on AD/ADRD, specifically on 2 platforms–Reddit’s r/Alzheimers and ALZConnected. For RQ2, we examined the prevalence and distribution of social support corresponding to these needs and concerns, and the association between these needs and received support.

**Methods:**

We collected 13,429 posts and comments from the r/Alzheimers subreddit spanning July 2014 to November 2023, and 90,113 posts and comments from ALZConnected between December 2020 (the community’s earliest post) and November 2023. We conducted topic modeling using latent Dirichlet allocation (LDA), followed by labeling to identify the major topical themes of discussions. We used transfer learning classifiers to identify the occurrences of emotional support (ES) and informational support (IS) in the comments (or responses) in the discussions. We built regression models to examine how various topical themes are associated with the kinds of support received.

**Results:**

Our analysis revealed a diverse range of topics reflecting community members’ varying needs and concerns of individuals affected by AD/ADRD. These themes encapsulate the primary discussions within the online communities: memory care, nursing and caregiving, gratitude and acknowledgment, and legal and financial considerations. Our findings indicated a higher prevalence of IS compared to ES. Regression models revealed that ES primarily occurs in posts relating to nursing and caring, and IS primarily occurs in posts concerning medical conditions and diagnosis, legal and financial, and caregiving at home.

**Conclusions:**

This study reveals that online communities dedicated to AD/ADRD support engage in discussions on a wide range of topics, such as memory care, nursing, caregiving, and legal and financial challenges. The findings shed light on the key pain points and concerns faced by individuals managing AD/ADRD in their households, revealing how they leverage online platforms for guidance and support. These insights underscore the need for targeted institutional and social interventions to address the specific needs of AD/ADRD patients, caregivers, and other family members.

## Introduction

Alzheimer disease and related dementias (AD/ADRD) consist of neurodegenerative conditions characterized by cognitive impairment, memory loss, and executive function decline [1]. Over 6 million individuals in the United States grapple with the challenges imposed by AD/ADRD [2], signaling a substantial and escalating public health concern, and underscoring the urgent need for comprehensive understanding and intervention. However, despite the existence of available resources, the long-term services and support for people with AD/ADRD exhibit inconsistencies within the existing health care system [3,4]. People with AD/ADRD and their caregivers often perceive interactions with others as reflective of patient infantilization and a lack of sensitivity and respect [5]. They frequently encounter added challenges, encompassing mental health issues, social exclusion, and instances of discrimination [6,7]. In fact, if caregivers’ concerns are overlooked, it may accelerate cognitive decline in individuals with AD/ADRD [8].

As technology gets more integrated into our lives and society, previous research has also noted the use of technology by AD/ADRD individuals and their caregivers [9]. Research highlights the significance of acknowledging and addressing the technology requirements and acceptance within AD/ADRD care [10]. However, more emphasis is placed on incorporating technology into AD/ADRD care, with the objective of improving the user experience to assist both patients and caregivers in managing daily life and monitoring their health status on a regular basis. A significant concern among AD/ADRD caregivers, however, remains the limited availability of adequate support systems at both institutional and social levels [11,12]. With the ubiquity of the internet, more individuals are turning to online spaces to seek information, discuss challenges, and share personal experiences. In particular, online communities are spaces that enhance a sense of belonging by connecting individuals with similar experiences. These online communities enable individuals to candidly self-disclose their concerns and seek support from others [13-20]. Previous work also noted how these online platforms could play a role in shaping the experiences of those living with progressive AD/ADRD [21-23]. These online support communities are very much in line with the social support behavioral theory (SSBC) that identifies the interactions intended to provide social support toward helping individuals cope with stress, enhance well-being, and foster positive relationships [24]. Nonetheless, social expectations and requirements in these online communities are largely unknown. Such knowledge would not only enhance our understanding of how the needs and concerns of AD/ADRD individuals and caregivers evolve over time but also help inform the design of targeted online interventions to address these needs.

This study aims to explore the needs and concerns of members within online communities on AD/ADRD, as well as the social support they actively provide and seek. To avoid any biases embedded within specific communities, we examined 2 distinct online communities—r/Alzheimers on Reddit and AlzConnected—dedicated discussion forums for AD/ADRD. Our study is guided by the following research questions (RQs): (1) RQ1: What are the needs and concerns people discuss in online AD/ADRD communities? (2) RQ2: What is the prevalence and distribution of social support across various needs and concerns, and how are different types of needs associated with the types of support received?

## Methods

### Data

We sourced our data from 2 distinct online AD/ADRD communities—r/Alzheimers on Reddit and AlzConnected, which we describe below:

#### Reddit

Reddit is a widely used semianonymous online platform that houses a network of over 52 million active users and 100 thousand active online communities. These communities are called “subreddits,”—each offering demographic, topical, or interest-specific discussion boards. The semianonymity of Reddit is known to enable candid self-disclosure and seek social support on stigmatized and sensitive topics [13,25,26], including discussions related to AD/ADRD [22,23,27].

We used Reddit’s search feature to look up subreddits related to AD/ADRD, and 2 of the largest subreddits returned were r/AlzheimersGroup (over 117 thousand members) and r/Alzheimers (over 13 thousand members). We then conducted sanity checks to understand the content of these subreddits. These investigations revealed that r/AlzheimersGroup was a community centered on humor and sarcasm, where people predominantly pretended to have forgetfulness and shared memes and cartoon references to Garfield [28]. r/AlzheimersGroup also self-describes that “Serious discussion belongs in r/Alzheimers,” Therefore, we omitted r/AlzheimersGroup from our ensuing analyses. The r/Alzheimers subreddit consisted of discussions where people shared troubles and provided social support on AD/ADRD. We collected all the posts and comments data from this subreddit—1000 posts and 12,429 comments between July 2014 and November 2023.

#### AlzConnected

AlzConnected is another freely accessible website (alzconnected.org) consisting of discussion boards dedicated to AD/ADRD patients and their caregivers. Although a more recent online community, it has already grown a significant user base and is supported by the Alzheimer Association (alz.org). Here, community members express AD/ADRD-related concerns and provide and seek support from one another. This community has multiple thematic discussion boards—(1) living with AD/ADRD, further broken down into “I am living with Alzheimer or other dementia and I am living with younger-onset Alzheimer,” and (2) Supporting someone living with AD/ADRD, further broken down into, “I am a caregiver (general topics), caring for a spouse or partner, caring for a parent, caring long distance, and supporting those who have lost someone.” We adopted web scraping approaches to collect the entire data from ALZConnected amounting to 10,328 posts and 79,785 comments from the community between December 2020 (earliest post in the community) and November 2023.

Table 1 summarizes our dataset from the 2 online communities.

**Table 1:** Summary of dataset from online communities catering to discussions on AD/ADRD^a^.

Community	Number of posts	Number of comments
r/Alzheimers	1000	12,429
ALZConnected	10,328	79,785

^a^AD/ADRD: Alzheimer disease and related dementias.

### Analyzing the Needs and Concerns

Toward answering RQ1, we conducted unsupervised topic modeling followed by human annotation to obtain the needs and concerns expressed by members of AD/ADRD online communities.

### Topic Modeling and Thematic Analysis

We started by examining the content of posts and comments in r/Alzheimers and AlzConnected to gain insights into the prevalent needs and concerns discussed within AD/ADRD online communities.

#### Building a Topic Model

Topic modeling techniques have been adopted to analyze and identify key themes within social media discourse [29-32] including AD/ADRD-related discussions [22,23]. We built topic models on our entire dataset (posts and comments) by using latent Dirichlet allocation (LDA) [33], an unsupervised machine learning algorithm widely used for analyzing corpora. LDA generates latent topic distributions, making it a valuable tool for analyzing large, unlabeled document collections and their clustering into distinct groups based on common themes. LDA has been heavily leveraged in previous work on social media language analysis [29,30]. After tokenization, stop word removal, and adding n-grams (n=2,3), we created bag-of-words representations of the dataset.

#### Determining the Optimal Number of Topics

Given that LDA does not automatically determine the optimal number of topics, we adopted a semiautomated approach, motivated by previous work [22,30,31]. For this, we first used the coherence score as a metric to assess the model fit, varying the number of topics. The coherence score evaluates the tendency of words within a topic to co-occur and correlates well with topic modeling quality [34]. Then, we hand-selected a few topic models that occurred in the zone of highest coherence scores. Two coauthors manually evaluated the quality of these different topic models in terms of assessing the between-topic heterogeneity and within-topic homogeneity of words to narrow down the best topic model for our ensuing analyses.

**Figure 1:**
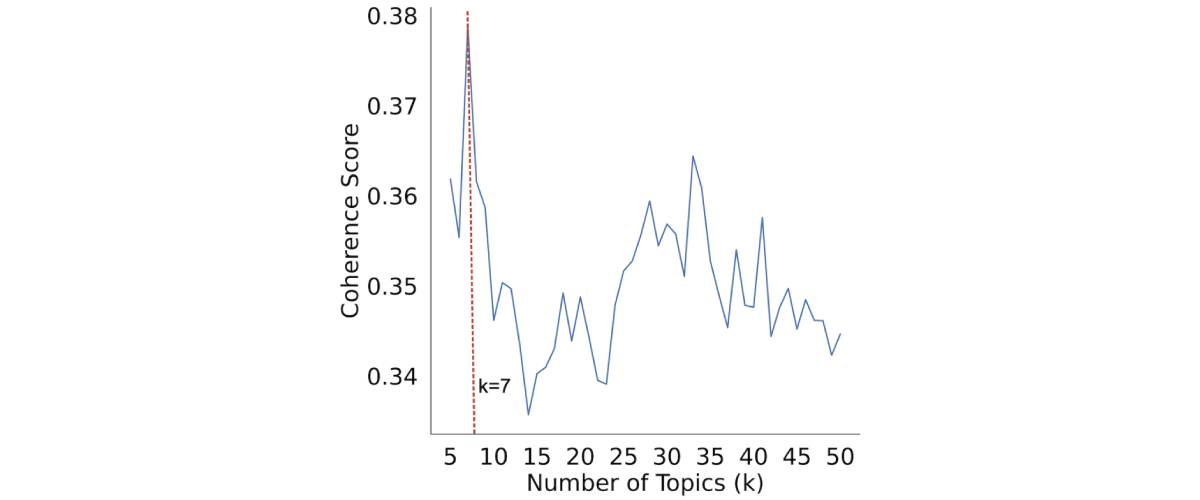
Coherence values over varying number of topics in the LDA (latent Dirichlet allocation) topic model.

We computed the coherence score for our 3 preprocessed corpora, varying the number of topics (k) from 5 to 50 (refer to Figure 1). We found the highest coherence at k=7. Consequently, we manually evaluated the quality of the topic modeling for k=5, k=7, and k=10.

#### Labeling the Topics

The above steps yielded topical clusters, each of which contained clusters of keywords. We adopted a thematic analysis approach to label each topical cluster in a meaningful and interpretable way. The thematic analysis was conducted by 2 authors, familiar with social media analysis and AD/ADRD literature. They adopted an inductive coding approach to first independently code the topics based on the cluster of keywords, referencing sample posts corresponding to each topic. Then, the authors engaged in collaborative discussions, comparing their codes, and gradually converging on cohesive thematic labels. This meticulous process ensured a nuanced and comprehensive analysis of the data. We found that the topic modeling with k=7 yielded the best results in terms of coherency within topics and boundaries between topics.

### Classifying Support and Examining Supportive Expressions

Toward answering RQ2, we analyzed the responses within the online communities to examine the nature and forms of social support provided. This section details our methodology for categorizing these responses into distinct subcategories and contrasts the patterns of support provision within the Alzheimer community against those observed in a broader user demographic.

### Classifying Support Expressions

#### Overview

The role of social support is essential in assisting individuals in managing psychological distress and various life adversities [35]. The emergence of social media platforms and virtual communities has led to the digital transposition of traditional social support mechanisms [13,36]. Specifically, online forums such as Reddit have increasingly mirrored the characteristics of virtual support groups, providing a platform for communal interaction and aid. The SSBC schema [37] offers a structured approach for classifying various types of support. Within this framework, informational support (IS) and emotional support (ES) emerge as 2 pivotal categories. Notably, these forms of support have garnered significant theoretical and empirical interest within social computing, as documented in various studies [20,38-43]. ES encompasses actions that provide encouragement, empathy, or care, whereas IS consists of assistance involving advice, information, or knowledge [37]. These 2 forms of support are particularly dominant in online interactions, as they can be effectively conveyed through text, making them well-suited for digital environments where individuals seek both guidance and emotional reassurance.

We categorized support offered in comments into ES and IS across both Reddit and ALZConnected datasets. To achieve this, we used a transfer learning methodology, a process wherein a machine learning classifier is developed by transferring insights from one labeled dataset to another unlabeled dataset with similar characteristics [44]. Specifically, we used a labeled Reddit dataset, previously expert-annotated with ES and IS labels on 396 comments collected from 55 mental health-related subreddits on Reddit [40]. This served as the seed dataset to train our classifiers. In addition, we manually labeled the ES and IS of 100 comments from AlzConnected and 100 comments from r/Alzheimers datasets and added these to the training datasets of the support classifiers. Our initial classifier was a supervised learning classifier for ES and IS, using n-grams (with n=1,2) as features in alignment with methodologies outlined in previous research [39-41]. We adopted a k-fold cross-validation (k=10) approach for evaluating our models.

**Figure 2:**
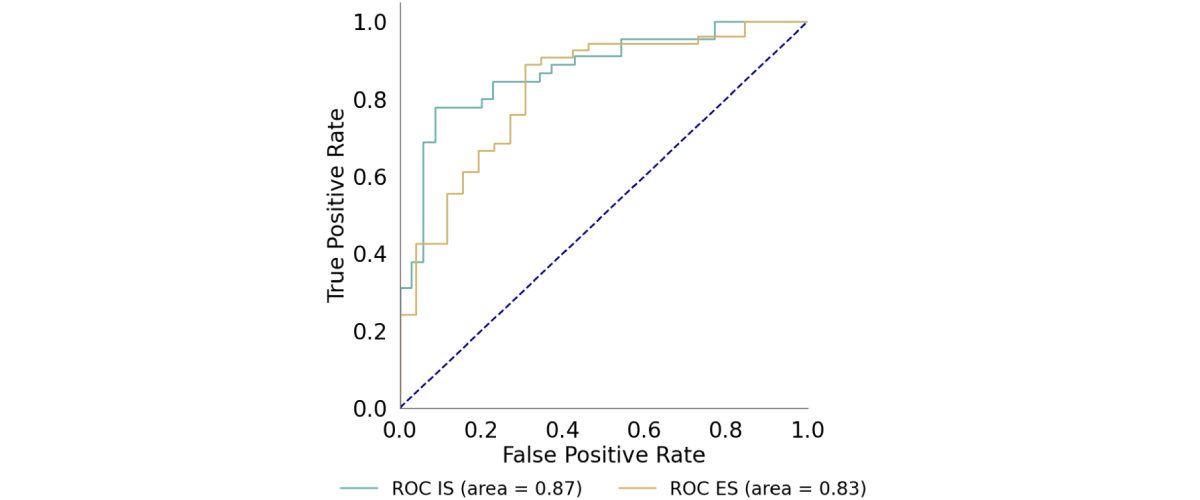
Receiver-operating-characteristic area-under-curve plot of the emotional and informational support classifiers. ES: emotional support; IS: informational support.

We used a range of classification algorithms, including naive Bayes, logistic regression, support vector machine (SVM), random forest, and neural network. Among these, the Linear SVM model demonstrated the best performance, achieving a mean area under the curve of 0.84 for ES and 0.87 for IS (refer to Table 2). Figure 2 provides the receiver-operating-characteristic area-under-curve plot for the classifiers used in identifying support types. We use the linear SVM model for our ensuing analyses. For the ES classifier, the predominant features include: “good,” “sorry,” “right,” “dont,” “going,” “people,” “get,” “life,” “im,” and “feel.” In contrast, the IS classifier highlighted features such as: “help,” “might,” “thing,” “go,” “think,” “try,” “get,” “see,” “probably,” and “doesnt.”

**Table 2:** Performance metrics of machine learning models of social support classifiers.

Model			Emotional support							Informational support				
			Precision		Recall		Area under the curve		Precision			Recall		Area under the curve
Naive Bayes			0.70		0.58		0.80		0.67			0.66		0.76
Logistic regression			0.73		0.69		0.76		0.75			0.76		0.81
Support vector machine			0.74		0.74		0.80		0.73			0.73		0.83
Random forest			0.68		0.54		0.75		0.67			0.66		0.75
Neural network			0.71		0.68		0.79		0.70			0.65		0.77
**After adding augmented labeled data from AD/ADRD^a^ communities**														
	Logistic regression	0.74		0.72		0.77		0.74			0.76		0.80	
	Support vector machine	0.75		0.76		0.83		0.77			0.78		0.87	

^a^AD/ADRD: Alzheimer disease and related dementias.

To validate the applicability and relevance of the model, we applied transfer-based learning to the classifiers within our dataset. We conducted a manual validation on a randomly selected subset of 100 comments. This process involved 2 independent annotators classifying each reply for ES and IS. Discrepancies in annotations were resolved through the intervention of a third adjudicator. Further, we compared these manual annotations with the predictions made by the machine learning classifiers. The comparison resulted in an *F*_1_-score of 0.76 for ES, characterized by a precision of 0.74 and accuracy of 0.76, and an *F*_1_-score of 0.77 for IS, precision of 0.77, and accuracy of 0.77, which is an increase from the initial classifier. Given these results, we consider the classifier’s performance satisfactory for further analysis. It is important to note that in our classification, ES and IS are not mutually exclusive categories; a single comment may be either both, or neither forms of support. Table 3 shows some example comments in our dataset with labels of ES and IS.

**Table 3:** Example comments and support labels with emotional and informational support (each comment may be either both, or neither forms of support).

Example comment	Emotional support	Informational support
Just as a note, when my Uncle passed away, I still remember him as the strong figure versus any thoughts of his Alzheimer. Man, my condolences and it’s more of a blessing for him and your family for the rapid decline.	✓	x
Lumbar punctures are great and the data is very solid in the ability to diagnose AD^a^. However, LPs can be lengthy (and therefore expensive) procedures and are generally viewed as invasive by the general population. A blood test such as this would highly impact our ability to screen people more quickly for drug trials :)	x	✓
Sometimes you just have to chuckle. I just received yet another call from my person with dementia telling me they needed someone to fix their phone. The same phone they made a call with. And we live over three hours away.	✓	✓
This feels like a lecture, which is not what I need right now. No reply is necessary.	x	x

^a^AD: Alzheimer disease.

#### Associating the Relationship Between Topics of Concern and Support Received

To assess the support associated with each topic, we used linear regression models to predict the likelihood of receiving different types of support. We built separate models for ES and IS. Each model was designed to predict the probability of receiving a specific type of support based on the topic being discussed. So, we used the topics in the posts’ content as independent variables, and the received support as the dependent variable. Essentially, by examining ordinary least squares regression models, we aimed to understand how different topics are associated with the probability of receiving a type of support. This approach allowed us to quantify the relationship between topics and the support they generate, providing valuable insights into the dynamics of support within the community.

### Ethical Considerations

This project used publicly available data from online communities, and did not involve any direct interaction with human subject participants. Therefore, this research did not require ethics board approval. However, we are committed to the ethics of the research and followed practices to secure the privacy of the individuals in our dataset. We recognize the sensitivities of our study in terms of revealing the identities of the individuals, and thus deidentified all collected data. This paper only presents paraphrased quotes to reduce traceability while providing necessary context.

## Results

### RQ1: Themes of Needs and Concerns in Online Communities

**Figure 3:**
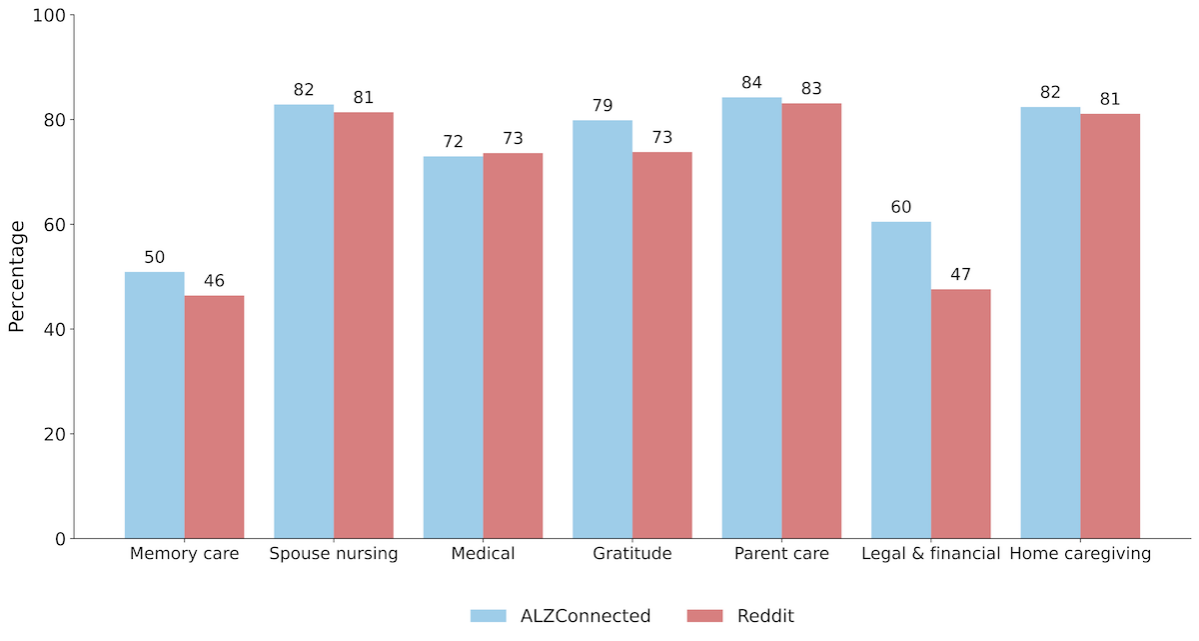
Distribution of topics as a percentage of posts on r/Alzheimers and ALZConnected.

We now describe the findings from our topic modeling followed by thematic analyses. Table 4 shows the 7 topics and relevant thematic analysis derived through LDA topic modeling. These topics serve as the primary themes, summarizing the needs and concerns of members within the AD/ADRD online communities. Figure 3 shows the overall distribution of topical occurrences in the 2 datasets, showing that the topics appear similarly on both platforms.

We describe these topical themes as: First, the topics reveal diversity in community members’ needs based on relationships with patients experiencing AD/ADRD. We find that several topics, such as memory care support, nursing for a spouse, caring for patients, and caregiving at home are linked to informal caregivers, such as patients’ spouses, children, and close family members, along with professional caregivers providing in-home assistance. This reveals that community members with different roles may encounter various concerns and needs.

We find a substantial amount of content focusing on memory care, particularly about early onset patients with AD/ADRD, with words like “memory care” and “assisted living facility” (Topic 1, 5, and 7). In general, memory loss is a common symptom of AD/ADRD [45]. Memory care is frequently observed as dedicated and free-standing memory care living spaces within assisted living communities or integrated into life care communities [46]. Individuals also discuss how to choose an assisted living home with a memory care unit for patients and issues encountered in memory care units. One individual posted in r/Alzheimers to seek suggestions:

Hello everyone, could we discuss the options for finding an assisted living facility with memory care? My wife has Early-onset Alzheimer, and she is 57.

We also find members who are early-stage patients looking for advice to plan the future and are gaining insight into experience within the memory care unit. An early onset patient posted:

Next week, I plan to visit an assisted living home with a memory care unit. My doctor has advised me to begin preparing for my future no matter what I can.

AD/ADRD leads to a progressive decline in the capacity to perform daily living activities [47]. We found extensive discussions related to nursing and care, with the majority originating from caregivers and patients’ family members (Topic 2, 5, and 7). Some content involves sharing AD/ADRD-related books and information. Within these posts, members recommend or summarize useful knowledge in a comprehensive way, and they are enthusiastic about discussing it with others. For instance, one caregiver posted their thoughts on the recommended book, “I recommend giving this book a read. Feel free to share your thoughts with me if you explore it.” Also, a significant amount of content revolves around the sharing of experiences and challenges. Some members shared experiences when they felt happy in a largely challenging journey of caregiving for their family members with Alzheimer. For instance, a member wrote;

He didn’t know who I was, but he got very excited that I helped him make handprint art to take to his 2 daughters and 3 granddaughters. He may not recognize me, but things like this show me the love is still there. Feeling blessed.

**Table 4:** Topic themes with representative keywords and example posts.

	Topic keywords	Example post
Topic 1: Memory care and assisted living	care, facility, memory, memory care, wife, home, living, darling, dear darling, dear, darling wife, dear darling wife, assisted, assisted living, nursing, staff, significant, living facility, assisted living facility, and room	*Is there anyone willing to share their experience of placing their loved one into a memory care unit?*
Topic 2: Nursing for spouse	significant, director, director nursing, nursing, just, husband, know, dear, time, like, day, good, dear husband, think, sorry, hope, things, did, going, and got	*If I get COVID, would my wife have to be placed in a nursing home? What actions should I do in such a situation?*
Topic 3: Medical condition and diagnosis	years, alzheimer, disease, husband, ago, alzheimer disease, significant, diagnosed, year, just, stage, diagnosis, early, time, years agodementia, months, know, care, and memory	*What support or resources are available for people in the MCI stage who may be unaware of it? I'm asking because my younger sister has been diagnosed with early-onset Alzheimer.*
Topic 4: Acknowledgment and gratitude	significant, thank, dementia, good, hospice, help, loved, support, meds, know, doctor, thanks, best, sorry, disease, helpful, forum, new, and information	*I'd like to thank those who recommended getting an electronic pet for a loved one with Alzheimer. I bought one for my grandma. She absolutely loves it!*
Topic 5: Caring for parents	mom, just, significant, like, time, help, know, mother, things, home, going, day, really, think, doesn, house, need, feel, dad, and tell	*I am an only daughter and I have taken on the responsibility of caring for my mother who received a diagnosis of Alzheimer and vascular dementia for 2 years.*
Topic 6: Legal and financial matters	attorney, husband, dear husband, care, dear, power, power of attorney, need, law, Medicaid, significant, help, elder law, money, long, state, law attorney, elder law attorney, and pay	*My husband was just diagnosed with younger onset Alzheimer. We are in search of an elder care lawyer in the Naperville or surrounding area to assist us with financial planning.*
Topic 7: Caregiving at home	dementia, dad, person, mom, family, care, make, person dementia, people, like, time, life, significant, need, help, things, think, caregiver, nursing, and home	*I have taken on the role of a “mom” for my mom; she relies on me for all her necessities, and needs, or just for chat. I’m only 20. I wish to live my own life. However, guilt keeps me at home, and I'm concerned that moving out might intensify it. Please tell me what to do.*

Some members place individuals with AD/ADRD in skilled nursing homes, while others serve as caregivers at home, leading to varying sets of issues. Some common concerns in nursing homes are the occurrence of living issues faced by patients residing in facilities, inappropriate interactions among patients, and instances where patients get expelled from the nursing facility and sent back home. We also found an instance where the patient required hearing aids, but the care provided by nursing facilities was not satisfactory. Consequently, a member posted:

Regrettably, there are many instances where the thing is either not carried out properly or not done at all. During my visits, I’ve observed that there are times when she has the hearing aids in, but they are non-functional.

In-home caregivers share the fatigue and strain arising from family relationships, as well as the distress of balancing their own lives with caregiving responsibilities. One member posted about being the sole caregiver without other siblings’ assistance. They wrote, seeking advice:

I have a full-time job and my own family. I find myself in need of a break, and I’m hesitant to consider placing her in a nursing home. I’m concerned that the unfamiliar environment might exacerbate her dementia.

Community members also expressed gratitude, encouragement, and acknowledgment. Members within online AD/ADRD communities can communicate about specific health-related topics, often finding online support a welcoming and comfortable venue for discussing sensitive issues [48]. Part of this involves mutual support and appreciation among community members, often occurring when someone shares their story or receives help and support from other members. Notably, posts and comments of this nature are typically longer and more detailed, prompting many individuals to express gratitude at the end, thanking others for taking the time to read their detailed posts. For instance, a member on r/Alzheimers, after venting about the challenges of being a caregiver, expressed:

Thanks for the opportunity to share my thoughts right here. I’ll read your many posts before mine and offer my opinions and encouragement.

Individuals also express gratitude not only directed toward other community members but also between caregivers and patients. Following the death of a loved one, members grieved recalling their shared past, the challenges of life, and caregiving after the diagnosis, such as, “I am thankful that his suffering finally ends. He is with my mom now in heaven.” Furthermore, when reminiscing about early shared experiences, members often express gratitude and love for the individuals with AD/ADRD, such as, “Reflecting on the cherished moments we shared brings me joy, and I am grateful for the knowledge he imparted to me.”

Finally, we also observed concerns related to legal and financial matters. Many of these concerns come from family members of individuals with AD/ADRD. With the progression of AD/ADRD, patients require increasing levels of care and assistance, leading to legal issues such as establishing guardianship or power of attorney (POA). Besides, financial concerns are a widely discussed topic in the communities, covering aspects such as handling patient’s assets, as well as the costs associated with caregiving and treatment. For example, a member posted to seek advice from the community:

Her funds is running down. We still hope that we can place her in a nursing facility after her funds are used up by the memory care (MC) facility. I need to know if there is any assistance available to support her continued stay in MC.

### RQ2: Social Support in Online AD/ADRD Communities

#### Distribution of ES and IS

**Figure 4:**
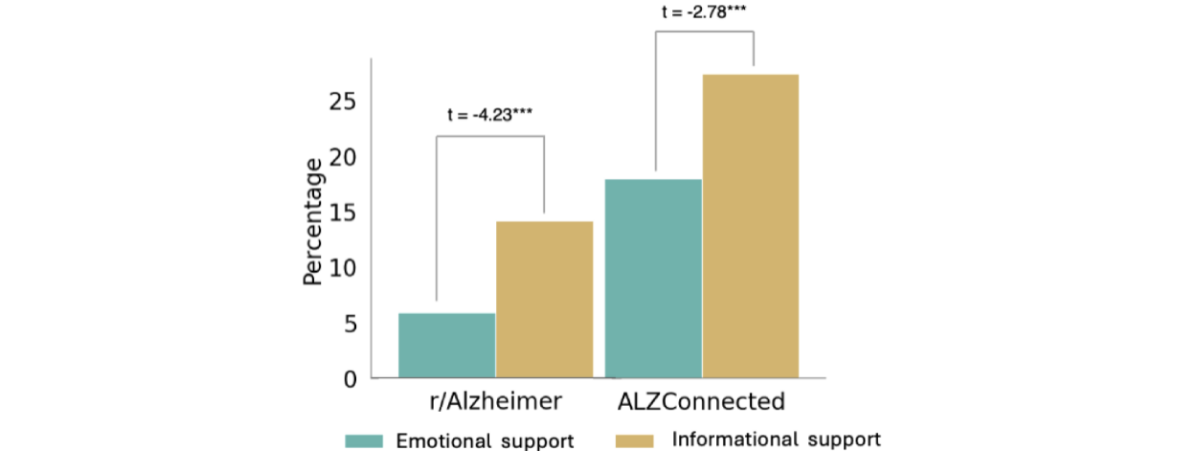
Distribution of ES and IS to posts on r/Alzheimers and ALZConnected.

We first examined how ES and IS are distributed in our datasets. We observe similar trends in the distribution of support in both communities—on average, IS is 6% more prevalent in r/Alzheimers and 10% more prevalent in AlzConnected than ES in respective communities. Figure 4 shows the overall distribution of ES and IS in the 2 datasets.

**Figure 5:**
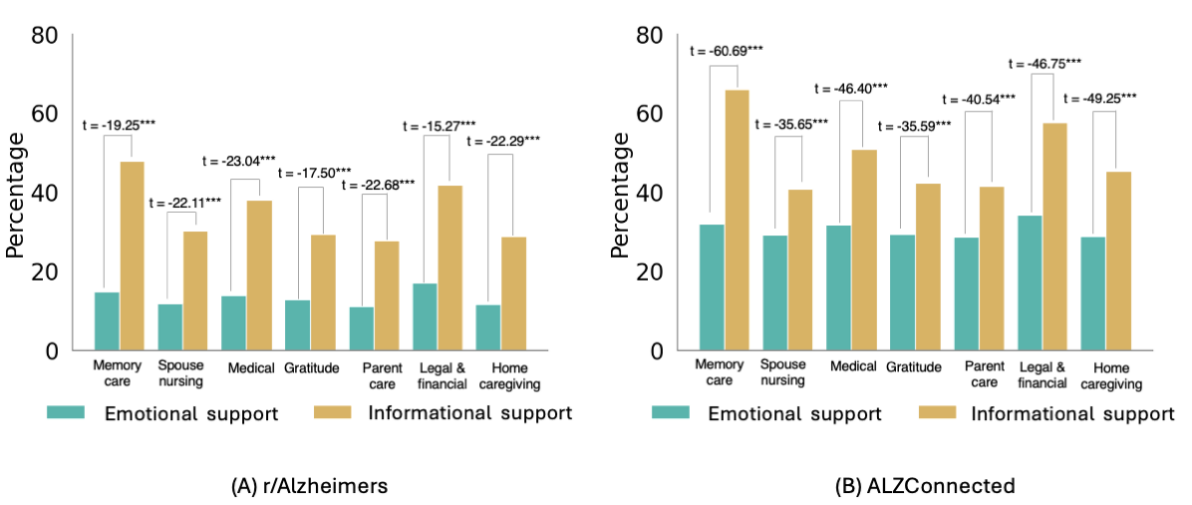
Distribution of emotional and IS in comments to posts with varying topics on (A) r/Alzheimers and (B) ALZConnected.

As previously noted, we obtained the topics on the posts and the support received in the comments to the posts. We break this down into the topics of the posts, and Figure 5 shows the distribution of ES and IS in the comments to the posts of varying topics in the 2 datasets.

Both Figures 4 and 5 also show independent sample *t* tests between the occurrences of ES and IS. For each topical theme, we note statistically significant differences in occurrences of the 2 types of support.

#### Regression Models for Predicting Support

To further analyze how support was provided corresponding to each topic, we built regression models. For each of our datasets (r/Alzheimers and AlzConnected), we built 2 models, 1 with IS as the dependent variable and another with ES as the dependent variable. Both of the models had all the topic occurrences as independent variables. Essentially, these models examined the likelihood of receiving ES and IS for each topic.

Table 5 shows the coefficients of the independent variables (topics) for ES and IS across the 2 datasets of r/Alzheimers and AlzConnected. All of these models show a significant goodness-of-fit (*R*^2^). These coefficients signal the likelihood of receiving the respective support in the community. We describe our observations below.

**Table 5:** Coefficients of linear regression models with the topics of posts as independent variables and support (emotional and informational) responses as dependent variables.

Independent variables	Dependent variables, coefficient			
	Emotional support		Informational support	
	r/Alzheimers	ALZConnected	r/Alzheimers	ALZConnected
Intercept	–3.00E-04	3.00E-04	–0.008^a^	0.027^a^
Memory care	0.014	0.012^b^	0.191^a^	0.258^a^
Nursing for spouse	0.016^b^	0.079^a^	0.066^a^	0.013^a^
Medical condition	0.048^a^	0.067^a^	0.182^a^	0.164^a^
Acknowledgment and gratitude	0.041^a^	0.058^a^	0.038^a^	0.034^a^
Caring for parents	0.014^c^	0.089^a^	0.031^a^	0.048^a^
Legal and financial	0.056^a^	0.075^a^	0.088^a^	0.13^a^
Caregiving at home	0.035^a^	0.038^a^	0.055^a^	0.129^a^
*R* ^2^	0.777^a^	0.144^a^	0.260^a^	0.294^a^

^a^*P*<.001.

^b^*P*<.01.

^c^*P*<.05.

#### ES Posts and Topics

For both r/Alzheimers and AlzConnected, we find that topics relating to nursing and caring, such as “Nursing for Spouse,” “Caring for Parents,” and “Caregiving at Home” show high positive coefficients with statistical significance, that is, posts with these topics are likely to receive ES. Further, topics that are seemingly about informational content, such as “Legal and Financial” and “Medical Conditions and Diagnosis” also receive ES.

For instance, an individual self-disclosed their stress about the fact that their mom changed a POA and was in complete denial of her symptoms:

I believe my mom just changed her POA and took me completely off. Her husband is covering for her and is in complete denial about her diagnosis. [..] She is clearly paranoid and delusional. I will consult with an elder care attorney soon. Any other advice? She blames me for her driver’s license getting taken away. I am the only family member in the state. Neither she nor her husband are capable of overseeing her medical care. [..] I am very stressed about this situation. Thank you for any thoughts.

#### IS Posts and Topics

For both r/Alzheimers and AlzConnected, posts relating to “Memory Care Support,” “Medical Condition and Diagnosis,” “Legal and Financial,” and “Caregiving at Home” show high positive coefficients with statistical significance. Interestingly, posts relating to “Nursing for Spouse” and “Caring for Parents,” which occurred high for ES, are also likely to receive IS. For instance, an individual who described their situation, also asked, “How are folks keeping their careers while helping to care for a parent?”

## Discussion

### Principal Results

This study sheds light on the complex needs of individuals dealing with AD/ADRD as patients, caregivers, family members, and loved ones. Our analyses revealed seven primary topical themes—(1) memory care and assisted living, (2) nursing for a spouse, (3) medical condition and diagnosis, (4) acknowledgment and gratitude, (5) caring for parents, (6) legal and financial aspects, and (7) caregiving at home. We found that IS was more prevalently provided than ES across both platforms. In particular, ES was predominantly found in posts related to nursing and caregiving, suggesting that caregivers turn to these communities to cope with the emotional burdens associated with their roles. In addition, IS was more frequently associated with concerns about medical conditions, legal and financial issues, and caregiving at home, indicating a demand for practical advice and resources in managing the complexities of AD/ADRD.

These findings highlight the critical role that online communities play in supplementing the support networks of AD/ADRD caregivers and family members. Many caregivers face challenges in accessing adequate in-person support due to stigma, social isolation, or lack of resources, making online platforms an important alternative for both emotional validation and practical advice—as also noted in the case of other stigmatized mental health [13,38-41,49] and health conditions (such as cancer [17,19,20] and HIV [50]). The predominance of IS suggests that individuals seek concrete advice to manage the daily realities of caregiving, including navigating health care systems, legal documentation, and financial planning. This aligns with previous research indicating that caregivers often experience a steep learning curve when managing AD/ADRD, requiring access to timely and accurate information [51,52]. Furthermore, the need for ES around nursing and caregiving emphasizes the significant emotional and psychological toll on caregivers [53]. These findings underscore the importance of community support in mitigating mental health issues such as feelings of burnout, stress, and isolation—some of the major concerns experienced by AD/ADRD caregivers [54]. We also noted expressions of gratitude and acknowledgment within these communities, which may indicate a sense of belonging and solidarity among the members of online communities, especially among caregivers to help boost their resilience and sustain their caregiving roles over the long term. Therefore, fostering these online communities can be a low-cost and scalable way to address caregiver burnout, which is a critical issue given the growing aging population and the increasing prevalence of AD/ADRD.

Our study also bears important implications for health care practitioners, policy makers, and support organizations. First, online communities provide empirical insights into the plausible gaps in accessible resources for caregivers. For instance, institutional support from governmental policies, health care systems, and AD/ADRD care organizations can leverage online platforms to identify the needs. Further, these platforms can be used to disseminate accurate and specific information tailored to the needs of the caregivers, potentially reducing the burden on healthcare professionals and improving caregiver confidence in managing AD/ADRD-related challenges. In addition, these platforms can be integrated into existing health care support structures and information.

Health care systems and AD care organizations could leverage online platforms to disseminate accurate, easily digestible information tailored to the needs of caregivers, potentially reducing the burden on health care professionals and improving caregiver confidence in managing AD/ADRD-related challenges. In addition, these platforms could be integrated into existing health care support structures, allowing caregivers to receive timely advice from health care professionals, thus improving patient outcomes. From a policy perspective, there is a need to enhance public health initiatives that support caregivers, especially in terms of financial planning and legal guidance, which was one of the major topics of discussion.

Policy makers could consider developing targeted programs that address these specific needs, such as offering workshops on financial planning for families affected by AD/ADRD or increasing access to legal assistance. Moreover, expanding caregiver support programs, including respite care and mental health services, could alleviate the emotional burden as prevalently discussed in the online communities. For technology designers and developers, our study suggests opportunities to enhance digital tools aimed at supporting caregivers. In particular, with the increasing prevalence of using artificial intelligence (AI) in different sectors, platforms can also incorporate AI-driven recommendation systems to provide personalized resources based on each individual’s specific question or concern. In addition, integrating mental health support tools, such as access to counseling services or peer support groups within these online communities, could further enhance the mental well-being of caregivers.

### Limitations

Our study has limitations which also suggest interesting future research directions. Although adopting computational approaches enabled our exploration of the depth and breadth of support dynamics across various topics, our findings are not necessarily generalizable to the broader population or other contexts beyond the online communities analyzed. As noted in Figure 3, although both communities follow the trend of having more IS posts than ES, the overall level of engagement across the 2 communities is not the same. In particular, our study is inherently limited by self-selection bias, as we only study individuals who actively chose to post and engage in these online communities. This means that our findings may disproportionately reflect the experiences of individuals who are more active on social media and are motivated and willing to share publicly on these platforms. In addition, this study does not capture the perspectives of passive users who browse forums for information without directly participating, which may exclude a significant portion of the online communities. Furthermore, digital inequity presents another challenge. Not all individuals have equal access to digital platforms, reliable internet, or the necessary skills to engage effectively online. This disparity can exclude voices from socioeconomically disadvantaged or digitally marginalized groups, leading to a biased understanding of support dynamics in online communities.

Accordingly, future research should explore strategies to mitigate these limitations by examining the representativeness of the user base and incorporating voices from underrepresented groups. For example, comparing findings from active participants with those of passive users or supplementing online data with offline sources could provide a more comprehensive view of support mechanisms. In addition, understanding how users of online support communities differ from those who might use AI-driven tools for AD/ADRD management is critical. Exploring the overlap and divergence between these groups will help inform the design of inclusive and effective digital interventions. Finally, the emergence of generative AI and large language models has enabled the development of personalized assistants and chatbots [55,56]. It would be interesting to examine how these AI chatbots respond to people’s queries on AD/ADRD and how effective they are compared to online communities.

### Comparison With Previous Work

Previous research has highlighted the significant burden imposed by AD/ADRD on patients, caregivers, and health care systems [54,57,58]. These caregivers often face stigma due to societal misconceptions about caregiving, which can result in judgment and disapproval, further intensifying their feelings of isolation and stress [59,60]. The growing demand for caregiving support due to the neurodegenerative progression of AD/ADRD necessitates increased levels of care over time that by 2030, an additional 1.2 million direct care workers will be required in the United States to address the rising needs of this population, pointing to a substantial gap in the current AD/ADRD caregiving infrastructure [2]. Our findings offer empirical evidence on the specific needs and concerns of individuals affected by AD/ADRD as they seek and exchange support within online communities.

Prior research has also underscored the psychosocial burdens faced by AD/ADRD caregivers, including feelings of marginalization and neglect of well-being [60,61]. A total of 69% of caregivers experience moderate to severe levels of caregiving burden, with the severity of the patient’s condition significantly impacting their stress levels [62]. Our study expands on this by underlining the specific concerns expressed in online communities and revealing that discussions frequently center on memory care, nursing, caregiving challenges, and legal and financial issues. This suggests that caregivers seek both practical advice as well as ES to manage the complexities of their roles.

A notable contribution of our research is its focus on the role of online platforms as a source of support for AD/ADRD caregivers. Previous studies have acknowledged the use of digital tools among caregivers [63] and highlighted a disconnect between the availability of digital support and the recognition of caregiving efforts within social circles [64]. Our findings on the major concerns and needs—as identified by topic modeling—also align with recent computational and qualitative analyses of Reddit and other online communities for AD/ADRD caregivers, which have identified similar themes, including ES-seeking, decision-making about care facilities, and management of behavioral symptoms [23,27]. Therefore, the integration of individuals with AD/ADRD and caregivers’ needs into the development of health and wellbeing technologies is necessary for future design [9,64]. Our study contributes to the recent body of work on the role of technologies in supporting caregivers—Bhat et al [65] emphasized the crucial role of caregiver-focused technological supports, and Kim et al [66] highlighted the need for tailored support strategies caregivers need during different phases of challenging behavioral episodes, and Meyerhoff et al [67] stressed the importance of user-centered digital mental health tools that adapt to individual needs.

The high prevalence of IS identified in our study aligns with existing literature that suggests caregivers often turn to digital platforms to fill gaps in health care services, particularly due to inconsistent coordination of long-term care [4]. The distribution of ES and IS across topics highlights a nuanced understanding of how caregivers use online communities to address specific facets of the caregiving experience. Aligning with how previous work focused on the use of technology for caregiving support [3,4], our study revealed the unique dynamics of social media as a tool for fostering a sense of belonging, solidarity, and social support among AD/ADRD caregivers. These observations align with findings from studies by Rains et al [68] and Naslund et al [69], who noted that individuals often leverage online spaces to combat loneliness and build supportive relationships. Together, our study builds upon the foundation laid by the SSBC, which categorizes support into 5 types—informational, emotional, esteem, tangible, and social network support [24]. Although this study focused specifically on ES and IS, we observed overlaps with other forms of support as well.

### Conclusions

This study reveals that online communities centered on AD/ADRD engage in extensive discussions on a diverse array of topics. These topics include memory care strategies, nursing and caregiving practices, as well as legal, financial, and emotional challenges associated with managing the disease. The findings highlight not only the primary concerns and pain points experienced by caregivers and families who manage AD/ADRD in their homes but also the critical role online platforms play in providing them with a sense of community, practical advice, and ES. By analyzing these discussions, the study identifies how individuals affected by AD/ADRD turn to online communities to share personal experiences, seek solutions to caregiving dilemmas, and obtain validation and reassurance from others who are navigating similar challenges. These online interactions can alleviate feelings of isolation, foster a collaborative environment for exchanging best practices, and serve as an essential resource for information and guidance. Our findings underscore the urgent need for targeted institutional and social interventions that address the nuanced and evolving needs of AD/ADRD patients, their caregivers, and their families. These interventions could include enhanced support services, caregiver education programs, and accessible mental health resources that are tailored to the specific challenges highlighted in these online discussions. By recognizing and addressing these needs, stakeholders can improve the quality of life for both patients and caregivers, ultimately contributing to a more supportive and inclusive environment for those affected by AD/ADRD.

## Abbreviations

AD/ADRD: Alzheimer Disease and Related Dementias
AD: Alzheimer Disease
AI: artificial intelligence
ES: emotional support
IS: informational support
LDA: latent Dirichlet allocation
POA: power of attorney
RQ: research question
SSBC: social support behavioral code
SVM: support vector machine
